# Timing of Surgery for Proximal Humeral Fracture Treated with Shoulder Hemiarthroplasty, Best Results with Surgery Within 2 Weeks

**DOI:** 10.1007/s43465-023-01079-y

**Published:** 2024-01-05

**Authors:** Yilmaz Demir, Alma Vuorinen, Max Gordon, Anders Nordqvist, Björn Salomonsson

**Affiliations:** 1 Orthopedic Department, Danderyd Hospital, 182 88 Stockholm, Sweden; 2 Karolinska Institutet, Stockholm, Sweden; 3 Skåne University Hospital, Malmö, Sweden

**Keywords:** Proximal humerus fractures, Arthroplasty of the proximal humerus, Surgery of the proximal humerus, Timing of surgery (or “time to surgery”), Shoulder hemiarthroplasty, Hemiarthroplasty, Proximal humerus

## Abstract

**Background:**

Preoperative delay may affect the outcome of proximal humerus fractures treated with shoulder hemiarthroplasty. There is currently no consensus for the recommended preoperative time interval. The aim was to examine how the time to surgery with shoulder hemiarthroplasty after a proximal humerus fracture affected the patient-reported outcome.

**Methods:**

380 patients with proximal humerus fractures treated with shoulder hemiarthroplasty recorded from the Swedish Shoulder Arthroplasty Registry were included. Three self-reporting outcome instruments were used at follow-up after 1–5 years: a shoulder-specific score, the Western Ontario Osteoarthritis of the Shoulder index (WOOS), the EuroQol-5 Dimension index (EQ-5D), and subjective patient satisfaction assessment.

**Results:**

The preoperative delay had a negative impact on the WOOS, EQ-5D, and patient satisfaction level (*p* < 0.01). The best result, measured with WOOS at a minimum 1-year follow-up, was found when surgery was performed 6–10 days after the reported date of fracture. WOOS% 8–14 days was 69.4% (± 24.2). A delay of more than 10 days was shown to be correlated with poorer outcomes. WOOS% 15–60 days was 55.8% (± 25.0) and continued to decrease.

**Conclusion:**

The current recommendation in Sweden to perform shoulder hemiarthroplasty within 2 weeks after sustaining a proximal humerus fracture is considered valid.

## Background

The proximal humeral fracture (PHF) is the third most common osteoporosis-related fracture in the population between 65 and 89 years and the prevalence is 4–6% [1–3]. The majority of PHFs are managed non-surgically [4]. There is no consensus regarding the surgical treatment for osteoporotic fractures in the proximal humerus and the decision is multifactorial.

Surgery with open reduction and internal fixation (ORIF) could be considered in the acute phase in dislocated PHF. The timing of surgery, with ORIF in the highly complex PHF, is suggested to be within 48 h to decrease the risk for avascular necrosis (AVN) [5].

Since the risk for failure is higher with ORIF in the elderly population, the alternative to ORIF is surgery with shoulder hemiarthroplasty (SHA) or reverse total shoulder arthroplasty (rTSA). Currently, the rTSA may be the most common choice [6, 7].

The primary indication for surgery with shoulder hemiarthroplasty is severely complex PHF on active patients with a high demand on load and work, good bone- and tendon quality, and with a good chance of healing of the tubercles [8]. Therefore, it is important to know the adequate timespan from trauma to surgery for the optimal decision based on the patient specific demand for the treatment.

Only a few studies have investigated the optimal timing for surgical treatment with a SHA for PHF, and the results of these suggest that early treatment is beneficial for the patient [9–11].

Based on clinical experience the current recommendation from the Swedish Shoulder and Elbow Society is proximal humerus fractures should undergo surgery within 14 days [12]. We hypothesized that the current recommendation would hold.

The primary aim is to determine the optimal time from trauma to surgery, related to outcome measured using the Western Ontario Osteoarthritis of the Shoulder Index (WOOS).

## Methods

This is a retrospective national cohort study. A total of 3383 shoulders were treated with shoulder hemiarthroplasty after proximal humerus fractures from the SSAR between 1999 and 2011. 2762 shoulders had an acute fracture diagnosis. We identified 1469 shoulders from a selection of nine hospitals, based on the willingness to participate in the study and the availability of medical records to confirm the date of trauma and Patient Reported Outcome Measures, PROM.

**Fig. 1:**
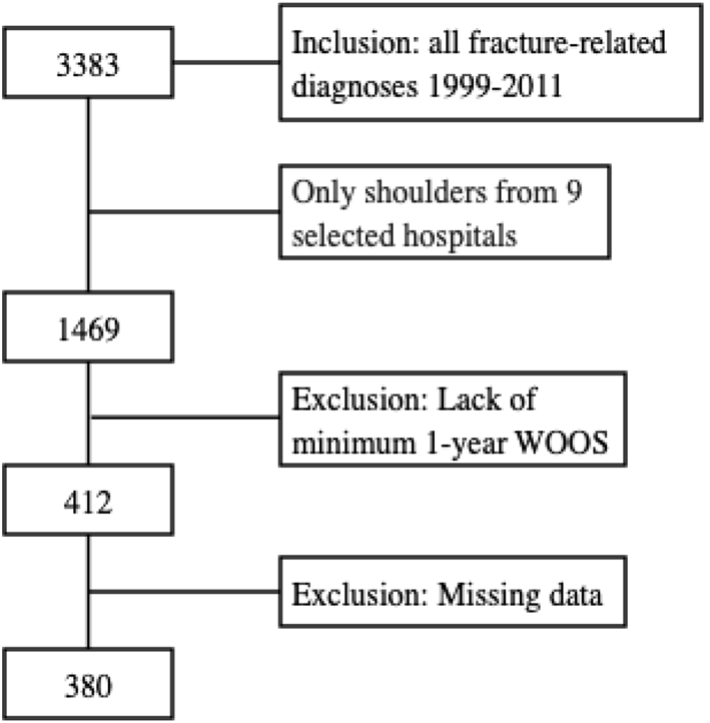
Flowchart of the inclusion of fracture-related arthroplasty procedures

Further exclusions were the lack of a minimum 1-year follow-up with WOOS until September 2012, the lack of a fracture date and missing data for the primary procedure left us with 380 shoulders, Fig. 1.

### Exposure

The exposure variable is the time from trauma until surgery, “timing to surgery”, the date of trauma was recorded in the medical records, and the date of the surgery was reported in the registry.

The time was then categorized into three groups: surgery—within 14 days, surgery—14–60 days, and surgery > 60 days after trauma. The day of surgery was also divided into weekdays and weekends.

The small subgroup surgery > 60 days was classified as “fracture sequelae” containing a mixed diagnosis defined as failed non-surgical treatment with malunion, nonunion, necrosis of the humeral head, or failed earlier surgery with an osteosynthesis when the first operation was over 60 days prior to revision with arthroplasty.

### Outcome

The primary outcome was a WOOS score measured at least 1-year after surgery. The secondary outcome was EuroQol-5 Dimension index (EQ-5D), and subjective patient Satisfaction level (SL).

### PROM

*WOOS* Consists of four domains [13]. Each of the 19 questions is answered using a visual analog scale. A total score of 0 is the best and 1900 is the worst possible outcome. This score is then converted into a percentage compared to a healthy shoulder. The minimal clinical important difference (MCID) is considered to be 10% [14].

*EQ-5D* Consists of five dimensions, mobility, self-care, daily activities, pain or discomfort, and anxiety or depression. Each combination of answers is represented by a specific total EQ-5D index [15].

*The satisfaction level* is one question regarding subjective overall satisfaction of the shoulder that had surgery. It’s a Likert scale with five steps; very dissatisfied, quite dissatisfied, neither satisfied nor dissatisfied, satisfied, and very satisfied.

We dichotomized the answers into two groups: the *dissatisfied group* with patients that were “quite dissatisfied” and “very dissatisfied”, and the *satisfied group* with all the other answers since an acceptable result is to avoid a dissatisfied patient.

### Statistics

Time to surgery was analyzed using linear regression models for WOOS% and the EQ-5D index, while logistic regression was used for satisfaction. In the first analysis, we excluded the fracture sequelae group. In the second analysis, we categorized “time to surgery” into three groups, “0–14 days”, “15–60 days”, and “> 60 days”. The two groups above 14 days also contained any surgery within the same timeframe defined as a fracture sequela.

To get a more detailed analysis of the “0–14 days” group it was further divided into “0–7 days” and “8–14 days”.

We omitted patients with incomplete measurements.

All regression models were adjusted for age, gender, day of the week for surgery, and the follow-up interval (5 years, and 1–4 years). Since the residuals for the linear regression models did not show a normal distribution, bootstrapping with 500 re-samples was used for inference.

The time was modeled using restricted cubic splines where the non-linearity was tested using analysis of variance (ANOVA) for each outcome [16]. The restricted cubic spline uses cubic terms in the center of the data but restricts the tails to straight lines as this has been shown to limit poor fits at the tails.

The flexibility of a spline is decided by the number of knots, we used 3 knots, as additional knots did not improve the Akaike Information Criteria (AIC). If the model indicated a non-linearity we investigated a possible cut-off point by using piece-wise linear regression with two lines [17].

By changing the smooth restricted cubic spline into two straight lines we could mimic a cut-off point situation without resorting to categories.

Values were calculated both in crude form and adjusted for the impact of co-variables to further analyze the statistical significance of the variables of interest. All analyses were performed with R v. 4.0.4, using the rms-package (v. 6.1–1) for modeling, knitr (v. 1.31) for reproducible research, and Gmisc (v. 2.0.1) for table output.

## Results

We studied 380 shoulders that had a minimum of 1-year follow-up with WOOS, EQ-5D, five-step Likert scale for satisfaction level and date of trauma. The study group consisted of 81% women with a range in age of 42–90 years (males age ranged 34–91 years). The majority (82%) had undergone surgery within 14 days after the trauma. The mean total WOOS% was 63% (± 26) and the mean EQ-5D index was 0.67 (± 0.3), Table 1.

**Table 1:** Baseline patient characteristics

Characteristic	*N* (%)	Mean age in years (SD)
Total study group	380	71 (± 11)
Female	308 (81)	72 (± 10)
Male	72 (19)	63 (± 12)
Dominant shoulder, right	321 (84)	
Fractured shoulder, right	201 (53)	
Surgery ≤ 14 days after trauma	287 (76)	71 (± 10)
Surgery 15–60 days after trauma	42 (11)	72 (± 11)
Fracture sequelae	51 (13)	67 (± 11)

### WOOS

We found a time-dependent impact on the total WOOS index for surgery within 60 days after trauma. The mean WOOS% for those treated within 0–7 days was 66% ± 25 and 69% ± 24 for the group 8–14 days. Surgery within 14 days scored higher than those with surgery later than 14 days after trauma, Table 2.

**Table 2:** Study population characteristics divided by days from trauma

Surgery after trauma	0–7 days	8–14 days	15–60 days	> 60 days/sequelae
N. patients	231	56	42	51
Age	71.2 (± 10.5)	70.7 (± 10.7)	72.0 (± 11.4)	66.5 (± 10.5)
Sex				
Female	190 (82.3%)	43 (76.8%)	33 (78.6%)	42 (82.4%)
Male	41 (17.7%)	13 (23.2%)	9 (21.4%)	9 (17.6%)
Day of the week for surgery				
Mon–Fri	215 (93.1%)	54 (96.4%)	41 (97.6%)	49 (96.1%)
Sat–Sun	16 (6.9%)	2 (3.6%)	1 (2.4%)	0 (0.0%)
Missing	0 (0.0%)	0 (0.0%)	0 (0.0%)	2 (3.9%)
Side				
Right	123 (53.2%)	30 (53.6%)	22 (52.4%)	28 (54.9%)
Left	108 (46.8%)	25 (44.6%)	20 (47.6%)	20 (39.2%)
Missing	0 (0.0%)	1 (1.8%)	0 (0.0%)	3 (5.9%)
Dominant shoulder				
No	96 (41.6%)	24 (42.9%)	18 (42.9%)	19 (37.3%)
Yes	105 (45.5%)	26 (46.4%)	19 (45.2%)	26 (51.0%)
Missing	30 (13.0%)	6 (10.7%)	5 (11.9%)	6 (11.8%)
WOOS%				
Mean % (SD)	66.2 (± 24.9)	69.4 (± 24.2)	55.8 (± 25.0)	48.2 (± 24.1)
Missing	10 (4.3%)	1 (1.8%)	2 (4.8%)	7 (13.7%)
EQ-5D index				
Mean (SD)	0.7 (± 0.3)	0.7 (± 0.3)	0.5 (± 0.3)	0.6 (± 0.4)
Missing	13 (5.6%)	1 (1.8%)	2 (4.8%)	8 (15.7%)
Satisfied				
No	57 (24.7%)	20 (35.7%)	18 (42.9%)	21 (41.2%)
Yes	169 (73.2%)	35 (62.5%)	24 (57.1%)	24 (47.1%)
Missing	5 (2.2%)	1 (1.8%)	0 (0.0%)	6 (11.8%)

Results in WOOS%, EQ-5D, and satisfaction level. Continuous variables are presented with mean and standard deviation

*EQ-5D* EuroQol-5 Dimension index, *WOOS* Western Ontario Osteoarthritis of the Shoulder index

**Fig. 2:**
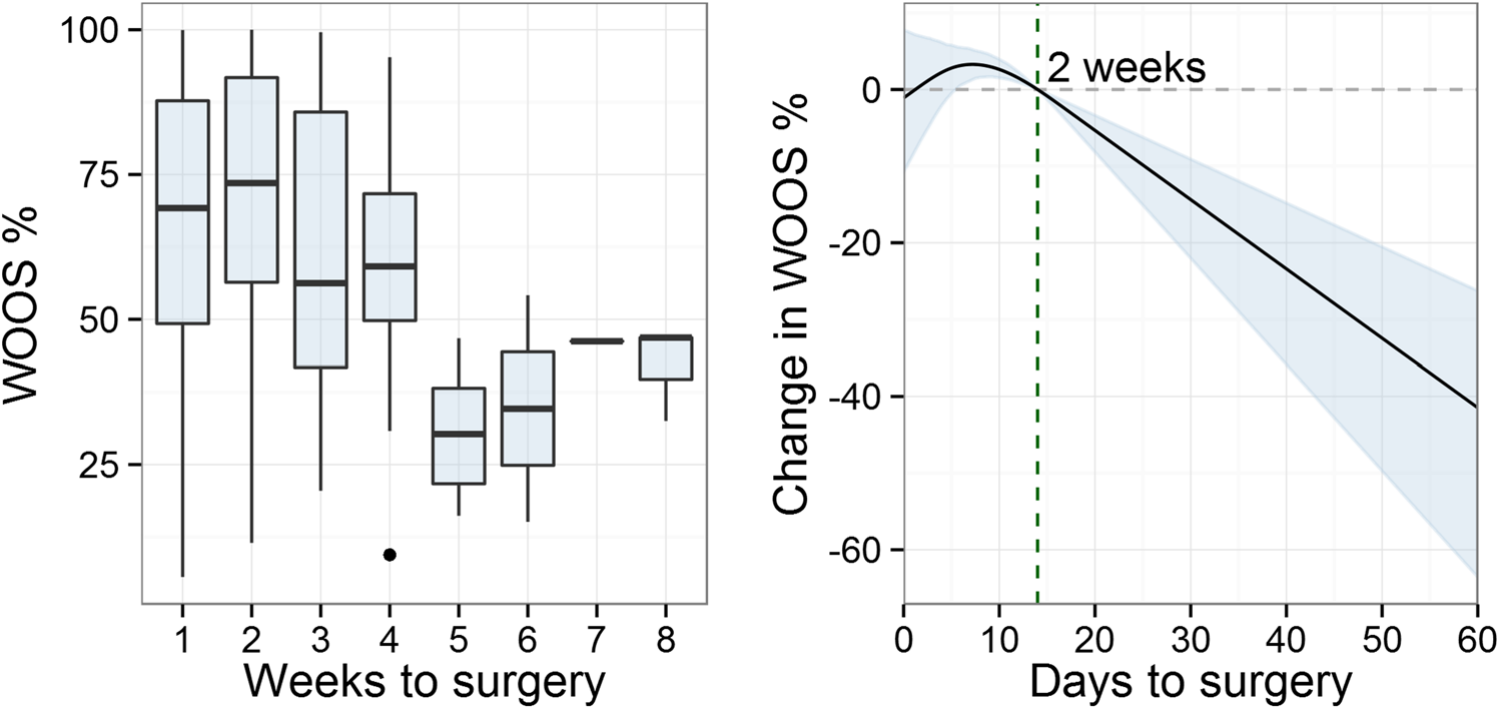
The outcome in WOOS% at minimum 1-year follow-up in relation to delay to surgery

Days to surgery showed a non-linear relation to WOOS%. The Surgery the first days after trauma showed an increase in WOOS% and reached a peak between days 6–10. The segmented model indicated a cut-off point at 10 days (95% CI 1–16 days) with a drop of -1.4 WOOS%/day after this cut-off point, Fig. 2.

The *left* plot is a boxplot with the median and 25th and 75th percentile for WOOS% divided into weeks from the fracture date. The *right* plot illustrates the outcome for each day of delay from trauma to surgery, with WOOS% on the y-axis, days on the *x*-axis, and 2 weeks as a reference point. The blue graph area indicates the 95% confidence interval.

### EQ-5D and Satisfaction Level After Surgery

For the EQ-5D index, the mean was 0.7 (± 0.3). Patients who had undergone surgery within 14 days after their trauma had a higher EQ-5D index than those 15–60 days after the trauma.

The proportion of dissatisfied patients was lower for those who had surgery within 14 days, 27%, compared to those who had surgery 14–60 days after the fracture, 45%, Table 2.

EQ-5D index had a linear decrease by − 0.03 per day (95% CI − 0.04 to − 0.02) and the satisfaction had a linear decrease with an odds ratio of 0.7 per day (95% CI 0.5–0.8).

### Subgroup Analysis

We found that “8–14 days” had slightly higher indexes compared to “0–7 days”, and “15–60 days” had worse scores in WOOS%, EQ-5D, and SL. There was a continued to decrease in the outcomes with WOOS% after 60 days but not EQ-5D and SL, Table 3.

**Table 3:** Comparison between the subgroups with WOOS, EQ-5D, and SL

	Linear regression		Logistic regression
	WOOS % (95% CI)	EQ-5D index (95% CI)	Satisfaction (OR) (95% CI)
Days to surgery			
0–7 days	0 (ref)	0.00 (ref)	1.0 (ref)
8–14 days	3 (− 4 to 10)	− 0.02 (− 0.06 to 0.02)	0.5 (0.3 to 1.0)
15–60 days	− 11 (− 19 to − 2)	− 0.09 (− 0.14 to − 0.05)	0.4 (0.2 to 0.9)
> 60 days or sequelae	− 17 (− 25 to − 10)	− 0.07 (− 0.12 to − 0.02)	0.4 (0.2 to 0.8)
Day of week for surgery			
Mon–Fri	0 (ref)	0.00 (ref)	1.0 (ref)
Sat–Sun	− 5 (− 15 to 4)	− 0.05 (− 0.09 to − 0.00)	0.3 (0.1 to 1.0)

0–7 days and weekdays are the references, and compared to those, the outcome is worse in all the other subgroups. This is represented either with a lower value or with a negative value

*CI* confidence interval, *EQ-5D* EuroQol-5 Dimension index, *OR* odds ratio, *WOOS* Western Ontario Osteoarthritis of the Shoulder index

WOOS % and the EQ-5D index are linear regression models while SL is based on a logistic regression model since its binominal.

A delay of 15–60 days after fracture demonstrated poorer outcomes in all three scores. Surgery during weekdays had better scores compared to surgery on weekends, Table 3.

## Discussion

Our findings show that surgery with SHA for PHF within 14 days of trauma had the best outcome with all the outcome scores. Poorer results with WOOS%, EQ-5D, and SL occurred after day 10; with a continued to decrease for each day passing. This supports the current guidelines that proximal humerus fractures should undergo surgery within 2 weeks [18, 19].

WOOS% also showed a slightly higher score, indicating an even better outcome, when surgery was performed a couple of days after the trauma as illustrated in Fig. 2. However, WOOS% has a 10% MCID [14] and the outcome is within a 95% confidence interval, which implies that there is no certain clinically significant benefit of surgical treatment with SHA within the first week.

The fracture sequelae group had a broad and scattered tail in Fig. 2 and it illustrates, that no matter the reason for surgery the results for very late surgery are significantly poorer than early surgery.

Compared to our findings some studies did not show time to surgery as a beneficial factor [20], still, they suggested that early surgery could be an advantage [21].

Our finding that early surgery is beneficial for the results is in concordance with several studies of “time to surgery” with less number of shoulders to analyze compared to our study [9, 11, 22–26].

The poorer outcome with surgery later than 14 days after trauma in this study may indicate that the healing process has already been initiated, leading to technical difficulties and impaired fixation and healing of the tubercles. Stiffness of the soft tissue could also be a complicating factor for delayed surgery [11]. Also for open reduction and fracture fixation of PHF, a delay of more than 5 days has been shown to increase the odds ratio for complications [27].

There is always a hierarchy of priority with trauma and ambulatory surgery. Since prior studies including our don´t show clinically significant benefits with surgery on the first days, we consider it safe to wait and do the surgery during office hours and weekdays if the proximal humerus fracture allows it and the patient is optimal for surgery.

There is time for the patient to assess their situation, and for the surgeon to optimize and re-evaluate the patient since some patients are uncertain of surgery or there are considerable medical risks with surgery.

Other important considerations in the decision to surgery are patient-related factors and the risk of falling again and they could weigh more than the fracture pattern [28]. Family and health workers can provide information that could increase the probability of the correct decision.

### Strengths

Previous studies consisted of less than 100 patients [9, 11, 20–22, 25, 29]. We had 380 patients collected from multiple centers to support our evidence.

A lot more patients had surgery with SHA after PHF in Sweden between 1999 and 2011 than are presented in this study. To ensure that we had a sufficient sample from SSAR, patients from nine hospitals that could provide data regarding the time of fracture were selected.

The result could be interpreted to be used in a generalized context and give valuable guidance to the clinician since all types of proximal humerus fractures were included when treated with SHA. We also had a follow-up period (1–5 years) that should indicate the final shoulder function after the initial rehabilitation phase.

The treatment with SHA after PHF is decreasing in Sweden and rTSA is increasing in the number of cases treated with arthroplasty [30].

### Limitations

The registry lacks information on the complexity, radiology before and after surgery, and classification of the fracture as this pertains from a cohort of patients who participated in a follow-up through the SSAR. The absence of pre- and post-surgery radiological data, which includes information about the state of the greater tubercle before surgery and the healing of the tubercles after surgery, could potentially impact the outcomes.

Furthermore, the type of fracture, the prior health status of the patient, the surgeon’s experience, and the choice of the prosthesis may have had an impact on the time to surgery and the outcome. This study aimed to examine the timing of surgery and not the treatment of individual fracture types.

The study exclusively relies on data from the Swedish population. Future studies could enhance the robustness of the findings by analyzing data from diverse countries.

Shoulder trauma rehabilitation is dependent on physiotherapy to optimize the range of movement, balance, and strength of the shoulder, but we lack information on the postoperative physiotherapy received. Data on the contralateral shoulder function for comparison was not available,

## Conclusion

The current recommendation not to delay a hemiarthroplasty more than 14 days after a proximal humeral fracture is supported by our findings. A few days of delay from the trauma does not seem to impair the result, as assessed by PROM. It is not necessary to perform the surgery immediately or outside of office hours.

## Data Availability

Data for this study is pseudonymized. The data is available upon request from the corresponding author, but with additional pseudonymization if required.

## Abbreviations

AVN: Avascular necrosis
EQ-5D: EuroQol-5 Dimension index
ORIF: Open reduction and internal fixation
PHF: Proximal humerus fracture
PROM: Patient-related outcome measures
rTSA: Reverse total shoulder arthroplasty
SHA: Shoulder hemiarthroplasty
SL: Satisfaction level
SSAR: Swedish Shoulder Arthroplasty Registry
WOOS: Western Ontario Osteoarthritis of the Shoulder index
